# Unifying two medicines to fight pain and to make anesthesia safer

**DOI:** 10.3389/fvets.2023.1248942

**Published:** 2023-09-04

**Authors:** Karine Portier

**Affiliations:** ^1^Université de Lyon, VetAgro Sup, CREFAC, Marcy l'Etoile, France; ^2^Université Claude Bernard Lyon, Centre de Recherche en Neurosciences de Lyon, INSERM, CRNL U1028 UMR5292, Trajectoire, Lyon, Bron, France

**Keywords:** algology, anesthesiology, veterinary medicine, comparative medicine, pain, animal

## Pain is a universal disease that affects both humans and animals

The International Association for the Study of Pain has defined pain as: “an unpleasant sensory and emotional experience associated with, or resembling that associated with, actual or potential tissue damage” (https://www.iasp-pain.org/publications/iasp-news/iasp-announces-revised-definition-of-pain/). The Association also states that the inability to communicate verbally does not prevent an individual from experiencing pain or appropriate analgesic treatment. Such statement brings the situation and condition of a non-communicative human patient close to that of an animal patient. The Guide for the Care and Use of Laboratory Animals also draws a parallel between human and animal pain and states that doubt should benefit the animal: “Unless otherwise established, researchers should consider that procedures that induce pain or stress in humans may cause pain or stress in other animals” (Principle 4, US Government Principles for the Utilization and Care of Vertebrate Animals Used in Testing, Research, and Teaching, https://olaw.nih.gov/policies-laws/gov-principles.htm).

Pain and its treatment in animals have been the subject of numerous publications over the last 30 years (1). In veterinary medicine, as in human medicine, associations have been created to combat pain. For example, the International Association for the Study of Pain (IASP) was created in 1974, whereas the International Veterinary Academy of Pain Management (IVAPM) was created in 2005. The goal of those associations is to promote, advance and improve pain management in animals. It is therefore now well-accepted that animals feel pain.

What seems to be obvious to us now, may have not been so evident in the 17th century. For example, Descartes, wrote in Discourse on the Method of Rightly Conducting One's Reason and of Seeking Truth in the Sciences in 1637: “The greatest prejudice of our childhood is to believe that animals think” (2).

For Cartesians, because animals could not think, they could not suffer, and it took several hundred years to modify such extreme belief. Jeremy Bentham wrote in 1789: “The question is not can they reason? Nor can they talk? But can they suffer?” (3).

Interestingly, this belief also applied to babies. Because they cannot use words to describe their sensations, it was believed that they could not feel pain. Forty years ago, conventional medicine still held that the neurological networks of newborns were immature and therefore under-functioning. Babies could not “feel” pain as adults could. Doctors were so convinced that newborns felt no pain that, until the mid-1980s, major surgeries (such as heart surgery) on newborns were sometimes performed without analgesia or even with neuromuscular blockers to prevent them from moving (4).

## Quantitative and qualitative importance of pain

While pain is not contagious, its prevalence is equal to more than one-fifth of the human and animal populations. If the exact number is difficult to assess in humans, it is even more so in animals. However, a few figures have been published. It is estimated that 20.4% (50.0 million) of American adults suffer from chronic pain (5). In France the prevalence of chronic pain in adults has been estimated to be between 27.2 and 32.7% (6). In dogs the prevalence of pain was estimated to be between 20% (7) and 38% (8).

Although not directly fatal, chronic pain prevents people from living. It has been identified as the fifth vital sign to be monitored in humans (9, 10). In veterinary medicine, the pain score has also been called the “fourth” vital sign, in addition to temperature, heart rate and respiratory rate (11). Freedom from pain is essential for welfare. The Farm Animal Welfare Council (FAWC) and the WOAH have published the fivee fundamental freedoms that define animal welfare, including freedom from pain, injury and disease.

For all these reasons, it is essential to make progress in pain management and to treat pain effectively, there is a need to recognize and gradate it. In both animals and humans, the major problem is to be able to assess pain objectively. Over the last 10 years, much progress has been made in the development of pain monitors (algometers), but most of these still need to be adapted and tested on animals. Exciting new discoveries on this subject are awaited.

## Treatment of pain and nociception is an integral part of the anesthesia protocol

Pain is of interest to most medical disciplines. In anesthesiology in particular, its prevention and treatment are part of the protocols. The European and American Colleges of Anesthesia have made algology their own adding an A to their name in 2013: European/American College of Veterinary Anesthesia and Analgesia: ECVAA, ACVAA.

Anesthetists use preventive anesthesia before surgery begins and the nociceptive stimulus is generated, they treat intraoperative nociception and manage postoperative pain. Effective treatment of pain and nociception before, during and after surgery is essential. Indeed, inadequate or excessive antinociceptive therapy leads to an increase in the length and cost of hospitalization and affects human patient recovery (12). Here too, studies are needed to assess the consequences of pain processing defects in animals.

The reported peri-anesthetic mortality is up to 100-fold higher than in human anesthesia (13). The current incidence and causes of anesthesia-related mortality in animals need to be reassessed (14). But we can assume that inappropriate perioperative pain management may have an impact on morbidity and mortality. Progress in the adjustment of analgesic treatment could therefore help to reduce the risk of anesthesia in animals.

## How to accelerate our progress in pain management and anesthesia risk reduction. The concept of comparative medicine, building bridges between human and animal medicines

Veterinary science is being repositioned at the heart of societal concerns. Animal health has become an essential and valuable biosecurity issue on a global scale. The international community has mobilized for global health around the “One Health” concept uniting major organizations. Major support of the “One Health” concept, comparative medicine is developing within human and veterinary clinics.

Indeed, pain research needs animal models (15). However, it is now known that so-called “laboratory” animal models have translational limitations (transfer between basic and clinical research) (16, 17). One of the reasons for this is that the so-called “laboratory animal” does not have any co-morbidities, unlike patients seen in a consultation.

Hence the importance of clinical comparative medicine, between patients, humans and animals with spontaneous pathologies (18). Natural spontaneous models of visceral pain (feline interstitial cystitis), neuropathic pain (diabetes in cats), osteoarthritis and cancer in several species have shown good translatability to humans (19).

The intensification of collaboration between human and animal medicines is necessary in algology. Indeed, studying the similarities and differences between humans and different animal species in order to establish a comparison of the causes, mechanisms and consequences of painful phenomena should make it possible to improve the effectiveness of the search for treatments.

In conclusion, there is a need to create and promote a space in which veterinarians and physicians could share experiences on pain and its treatment. Publications in the interface of both medicines, jointly conceived and authored by experts in the fields of algology and anesthesiology, are welcome and needed. When human and veterinary medicine join forces, the strike force of science will outweigh that of pain, our common “enemy”.

## Author contributions

The author confirms being the sole contributor of this work and has approved it for publication.

## References

[1] Stafford KJ Mellor DJ. Pain: a developing issue in veterinary medicine. Vet J. (2007) 174:225–6. 10.1016/j.tvjl.2007.03.020 17513146

[2] Descartes R Veitch J. A Discourse on Method: Meditations on the First Philosophy Principles of Philosophy. London: The Everyman Library (1994).

[3] Bentham J. An Introduction to the Principles of Morals and Legislation. Mineola, NY: Dover Publications (2007).

[4] McGrath PJ. Science is not enough: the modern history of pediatric pain. Pain. (2011) 152:2457–9. 10.1016/j.pain.2011.07.018 21880428

[5] Dahlhamer J Lucas J Zelaya C Nahin R Mackey S DeBar L. Prevalence of chronic pain and high-impact chronic pain among adults — United States, 2016. Morb Mortal Wkly Rep. (2018) 67:1001–6. 10.15585/mmwr.mm6736a2 30212442 PMC6146950

[6] Chenaf C Delorme J Delage N Ardid D Eschalier A Authier N. Prevalence of chronic pain with or without neuropathic characteristics in France using the capture–recapture method: a population-based study. Pain. (2018) 159:2394–402. 10.1097/j.pain.0000000000001347 30028790

[7] Muir WW Wiese AJ Wittum TE. Prevalence and characteristics of pain in dogs and cats examined as outpatients at a veterinary teaching hospital. J Am Vet Med Assoc. (2004) 224:1459–63. 10.2460/javma.2004.224.1459 15124886

[8] Rousseau-Blass F O'Toole E Marcoux J Pang DSJ. Prevalence and management of pain in dogs in the emergency service of a veterinary teaching hospital. Can Vet J. (2020) 61:294–300. 32165754 PMC7020628

[9] Lanser P Gesell S. Pain management: the fifth vital sign. Healthc Benchmarks. (2001) 8:68–70. 11474948

[10] Morone NE Weiner DK. Pain as the fifth vital sign: exposing the vital need for pain education. Clin Ther. (2013) 35:1728–32. 10.1016/j.clinthera.2013.10.001 24145043 PMC3888154

[11] Gruen ME Lascelles BDX Colleran E Gottlieb A Johnson J Lotsikas P. 2022 AAHA pain management guidelines for dogs and cats. J Am Anim Hosp Assoc. (2022) 58:55–76. 10.5326/JAAHA-MS-7292 35195712

[12] Oderda GM Gan TJ Johnson BH Robinson SB. Effect of opioid-related adverse events on outcomes in selected surgical patients. J Pain Palliat Care Pharmacother. (2013) 27:62–70. 10.3109/15360288.2012.751956 23302094

[13] Carter J Story DA. Veterinary and human anaesthesia: an overview of some parallels and contrasts. Anaesth Intens Care. (2013) 41:710–8. 10.1177/0310057X1304100605 24180711

[14] Gent TC Bettschart-Wolfensberger R. Peri-anaesthetic mortality in horses - the need for CEPEF-4. Vet Anaesth Analg. (2013) 40:e1–2. 10.1111/vaa.12070 23869801

[15] Mogil JS Davis KD Derbyshire SW. The necessity of animal models in pain research. Pain. (2010) 151:12–7. 10.1016/j.pain.2010.07.015 20696526

[16] McGonigle P Ruggeri B. Animal models of human disease: challenges in enabling translation. Biochem Pharmacol. (2014) 87:162–71. 10.1016/j.bcp.2013.08.006 23954708

[17] Mogil JS. Animal models of pain: progress and challenges. Nat Rev Neurosci. (2009) 10:283–94. 10.1038/nrn2606 19259101

[18] Alder M Easton G. Human and veterinary medicine. BMJ. (2005) 330:858–9. 10.1136/bmj.330.7496.858 15831850 PMC556144

[19] Klinck MP Mogil JS Moreau M Lascelles BDX Flecknell PA Poitte T. Translational pain assessment: could natural animal models be the missing link? Pain. (2017) 158:1633–46. 10.1097/j.pain.0000000000000978 28614187

